# Get a grip—evolution of claw shape in relation to microhabitat use in intertidal arthropods (Acari, Oribatida)

**DOI:** 10.7717/peerj.8488

**Published:** 2020-02-13

**Authors:** Tobias Pfingstl, Michaela Kerschbaumer, Satoshi Shimano

**Affiliations:** 1 Institute of Biology, University of Graz, Graz, Austria; 2 Science Research Center, Hosei University, Tokyo, Japan

**Keywords:** Geometric morphometrics, Ecomorphology, Semilandmarks, Adaptation, Littoral

## Abstract

Claws may be the most common biological attachment devices in animals but relatively few studies have examined the ecological and evolutionary significance of their morphology. We performed the first geometric morphometric investigation of arthropod claws using 15 intertidal oribatid mite species from two different families living in three different habitat types to determine if claw shape is correlated with ecology. Our results show that species living on rocky shores show remarkably high and strongly curved claws while species from mangrove habitats show significantly lower and less curved claws. Euryoecious species are able to dwell in a wide range of habitats and show an intermediate claw type. These results indicate a strong relationship between claw shape and microhabitat and the best predictors of microhabitat use seem to be claw height and curvature. Claw length varied to some degree among the species but without any noticeable ecological pattern. A comparison with terrestrial and freshwater aquatic oribatid mite species, on the other hand, confirms that their claws are only half as long as that of intertidal mites and it is suggested that tidal flooding and wave action strongly selects for long claws. In this microarthropod group which occupies a vast array of microhabitats, claw morphology may play an important role in niche separation and hence demonstrate the importance of ecomorphological studies.

## Introduction

Claws can be found in nearly all animal groups, from tiny tardigrades to large mammals, and they basically represent the first and last contact with the substrate during locomotion (Birn-Jeffery et al., 2012). Hence, claws may be the most common biological attachment devices (Zani, 2000), they can also fulfill various other functions, as for example climbing, digging and grasping prey (Tinius & Russell, 2017). Claw characteristics, such as curvature, length and height, are important parameters related to behavior and feeding strategy (Ribas et al., 2004) and thus were studied in several taxa to infer correlations between morphology and ecology. These studies indicated, for example, that claws of arboreal birds are more curved than those of ground-dwelling birds (Burnham et al., 2011) or that predatory birds exhibit more tapered claws with a more marked curvature than non-raptorial birds (Csermely, Rossi & Nasi, 2012). In lizards, arboreal species also show a greater degree of curvature and a greater height of the claw than typical terrestrial species (Zani, 2000; Crandell et al., 2014), and similar patterns can be found in mammals when comparing arboreal versus terrestrial habitats (Manning et al., 2006). These studies demonstrated that the basic design of vertebrate claws is similar among groups allowing for cross-species comparisons (Zani, 2000). Therefore, claw characteristics have been frequently used as an indicator of the ecology in fossil reptiles, for example, Manning et al. (2006) proposed that the hypertrophied claw of the predatory dinosaur *Deinonychus* was most likely used as a climbing crampon and not as a slashing weapon, as previously supposed. Another study (Feduccia, 1993) suggested that the widely known and famous *Archaeopteryx* was a perching bird because it exhibits a claw curvature typical of perching and tree-climbing birds.

All these investigations, correlating claw morphology with habitat and lifestyle, were conducted almost exclusively in vertebrates (Pattrick, Labonte & Federle, 2018) and invertebrates remained underexplored though their majority possesses claws. The few existing studies showed that certain insects can hold prey more effectively if the claw is more curved (Petie & Muller, 2007) and that attachment ability of small arthropods is basically determined by claw tip diameter and substrate roughness (e.g.,  Dai, Gorb & Schwarz, 2002; Heethoff & Koerner, 2007; Pattrick, Labonte & Federle, 2018), i.e., sharper claw tips will have more asperities to interlock with but are also more likely to break (Labonte & Federle, 2015). Another study unexpectedly demonstrated that a tiny soil-dwelling oribatid mite species produces exceptionally high relative claw forces probably topping all other organisms (Heethoff & Koerner, 2007). Apart from these few functional studies, insect and chelicerate claws have been mostly neglected, especially in terms of ecomorphology.

Mites (Acari) represent the smallest chelicerates and are thought to walk ‘through’ rather than ‘over’ a landscape, i.e., the landscape on this microscopic scale is highly structured and uneven terrain. Considering the wide range of ecological niches occupied by these tiny organisms, the landscape microstructure is highly variable for the different ecological groups (Heethoff & Koerner, 2007). Therefore, studying their claws may provide important insights into the correlation between morphology and ecology and may unravel their role in niche separation. Mites use their claws only for attachment and show different claw character states from three-claws (tridactyl), which are supposed to be plesiomorphic (Dunlop, 2002), to a single claw on each leg (monodactyl). These claws are formed of modified setae and are moved through the action of only two muscles, a levator and a depressor, which connect by tendons to a basilar piece containing the claws (Grandjean, 1941).

With regard to a possible relationship between phenotype and ecology, there was only a single study on oribatid mites from a mangrove forest showing that the number of tridactyl species was higher in the canopy and trunk while the root and littoral zone was dominated by monodactyl species. The relative length of this single claw was significantly larger in the latter environments and was suggested to be a consequence of selection for regular tidal flooding requiring the mites to grip tighter on the substrate (Karasawa & Hijii, 2004). Though the above-mentioned study demonstrated a correlation between macrohabitat and relative length of claws, it remained unclear if shape, curvature and sharpness also somehow correlate with ecology. Littoral monodactyl oribatid mites feed and dwell in intertidal algae growing on a wide variety of substrates, as for example rocks, concrete, mud, deadwood or mangrove roots (e.g.,  Pfingstl, 2017). Considering the diversity of these substrates and given the assumption that tidal flooding and wave action cause strong selection in terms of movement and attachment, we hypothesized that claw shape of littoral oribatid mites correlates with microhabitat, substrate use respectively. A modern technique for measuring shape variation in biological structures is geometric morphometrics (GM). It is based on the statistical analysis of landmark coordinates (Bookstein, 1991; Adams, Rohlf & Slice, 2004; Slice, 2007; Mitteroecker & Gunz, 2009). Semilandmarks make it possible to quantify two or three-dimensional homologous curves and analyze them together with traditional landmarks (Gunz & Mitteroecker, 2013). Here, we performed the first geometric morphometric investigation of arthropod claws ever using oribatid mite species belonging to the exclusively intertidal and monodactyl Fortuyniidae and Selenoribatidae. Our aims were (1) to assess variation in claw morphology, (2) to determine whether claw features correlate with ecology, and (3) to describe the differences in claw design.

## Material and Methods

For the investigation of claw shape and size in littoral oribatid mites, we analyzed 169 specimens from 15 different species. Samples were collected on several field trips to various geographic regions (details of sample locations are given in Table 1). Species in our study belong to two different marine associated families, namely the Fortuyniidae and Selenoribatidae. Fortuyniid species are *Alismobates galapagoensis*, *A. inexpectatus*, *A. reticulatus*; *Fortuynia atlantica*, *F. hawaiiensis*, *F. rotunda*, *Litoribates bonairensis*, *L. caelestis* and *L. floridae*; selenoribatid species are *Carinozetes bermudensis*, *C. mangrovi*, *Thalassozetes balboa*, *T. barbara*, *Thasecazetes falcidactylus* and *Thasecazetes* sp. Based on literature data (Pfingstl, 2015; Pfingstl, Lienhard & Jagersbacher-Baumann, 2014; Pfingstl et al., 2017) and personal observations, the ecology of these species was assessed and classified into three microhabitat types, (I) ‘rock’: all occurring exclusively on algae growing on rocky substrate, like boulder cliffs and man-made concrete structures, (II) ‘mangrove’: only found in mangroves, either dwelling in algae on the roots or in leaf litter, and (III) ‘mix’: euryoecious, meaning they equally inhabit algae on rocks and on mangrove roots and they also occur in algae on other soft substrates like muddy intertidal soils. A list of all intertidal species and their ecological classification is given in Table 1.

**Table 1:** Sample information. Information about sample location, sample year, ecology, habitat and number of individuals in different analysis for each mite species.

**Species**	**Sample origin**	**Date**	**Ecology**	**Habitat**	***n***	**n (CL/BL)**
*Alismobates galapagoensis*	Galapagos	1987	littoral	mix	9	8
*Alismobates inexpectatus*	Bermuda	2011	littoral	rock	25	18
*Alismobates reticulatus*	Japan	2019	littoral	mix	12	12
*Carinozetes bermudensis*	Bermuda	2011	littoral	mix	11	10
*Carinozetes mangrovi*	Bermuda	2011	littoral	mangrove	7	6
*Fortuynia atlantica*	Bermuda	2011	littoral	rock	23	20
*Fortuynia hawaiiensis*	Hawaii	1994	littoral	rock	8	7
*Fortuynia rotunda*	Japan	2019	littoral	mangrove	13	7
*Litoribates bonairensis*	Bonaire	2016	littoral	mangrove	18	15
*Litoribates caelestis*	Galapagos	1986	littoral	mix	10	9
*Litoribates floridae*	Florida	2017	littoral	mangrove	7	7
*Thalassozetes balboa*	Panama	2017	littoral	rock	10	8
*Thalassozetes barbara*	Grenada	2016	littoral	rock	8	8
*Thasecates falcidactylus*	Bonaire	2016	littoral	mangrove	3	3
*Thasecazetes* sp*.*	Florida	2017	littoral	mangrove	5	5
*Dolicheremaeus dorni*	Austria	2017	terrestrial	dead wood	7	7
*Mycobates parmeliae*	Austria	2018	terrestrial	mosses, trees	10	10
*Hydrozetes lemnae*	Austria	2017	aquatic	freshwater	12	12
*Hydrozetes* sp.	Turkey	2016	aquatic	freshwater	4	4

As outgroups we analyzed the claws of two typical terrestrial monodactyl species, *Mycobates parmeliae* and *Dolicheremaeus dorni* (we had several specimens in our collection that we could use immediately for the study and these two taxa show a wider ecological range and thus we could exclude a bias caused by occurring in strict terrestrial ecological niche) and two aquatic species (living in freshwater habitats), *Hydrozetes* sp. and *Hydrozetes lemnae*. For those species we defined no microhabitats (i.e., type of substrate). They are listed as ‘terrestrial’ and ‘freshwater’ (Table 1) in order to check for differences in shape and size of their claw compared to littoral species. Including outgroups, claws of 202 mites from 19 different species living in five different habitats/substrates were investigated.

In this study, we used a balanced number of males and females in most species and preliminary data did not show specific differences between the sexes. Therefore, we pooled both sexes and did not further separate them in our analyses.

**Figure 1:**
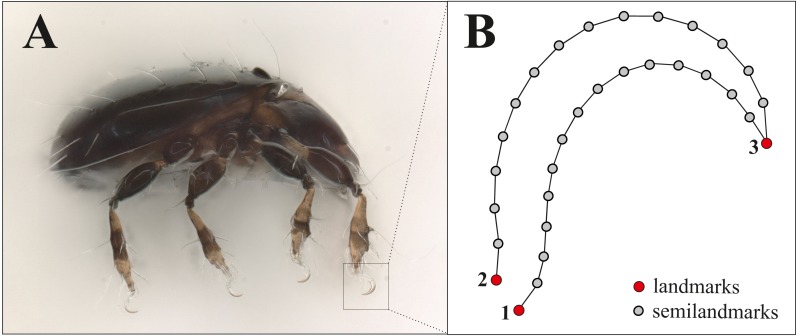
Landmark positions on the claw. (A) Habitus of Fortuynia atlantica and (B) schematic drawing of the claw of leg I. Red dots are landmark positions, grey dots represent positions of semilandmarks.

For doing claw morphometrics, each specimen was embedded in a microscopic slide using lactic acid and then they were photographed in dorsal and lateral view with a digital microscope (Keyence VHX-5000). Afterwards, they were crushed by applying pressure so that the remaining legs with the claws were caught in a lateral position between object carrier and object slide. As there are no apparent differences between the claws of the different legs of a single specimen and for standardization, we photographed and analyzed the claw of the first leg only. From all those photographs body length and claw length (after Fig. 1 in Karasawa & Hijii, 2004, p. 385) were measured with VHX-5000_900F-Datenkommunikationssoftware Version 1.6.0.0. The *x*, *y* coordinates of three landmarks (LM) and 32 semilandmarks were recorded using TpsDig2 (Vers.2.31, Rohlf, 2017). Figure 1 shows the positioning of landmarks and semilandmarks. Dorsally between landmarks 2 and 3, and ventrally between landmarks 1 and 3, 16 semilandmarks were placed equidistant along the claw edges. For the analysis we removed four semilandmarks reflecting nearly the same positions as LM 1-3. This resulted in three landmarks and 28 semilandmarks, the latter of which were slid to minimize the bending energy (Gunz & Mitteroecker, 2013) using TpsRelw Vers.1.76, Rohlf, 2018). Raw data with all measurements and landmark coordinates are provided in the supplemental files, photographs of the claws were uploaded to the internet repository Dryad (DOI https://doi.org/10.5061/dryad.0gb5mkkxc).

Six of the investigated littoral mite species (*Carinozetes bermudensis*, *C. mangrovi*, *Thasecazetes falcidactylus*, *Thasecazetes* sp., *Thalassozetes balboa* and *T. barbara*) exhibit a minute proximal ventral tooth on their claws which has been ignored in landmark analysis. To gain information about size differences, we compared body length and ratios of body length to claw length among different habitats and species. We used PAST, PAlaeontological STatistics 3.26 (Hammer, Harper & Ryan, 2001) for the standard statistical analyses and graphical representation. Analyses of shape data was conducted with MorphoJ (Klingenberg, 2011) and PAST (Hammer, Harper & Ryan, 2001). Because size is known to influence shape through ontogenetic stages and allometric trajectories (Cavalcanti, 2004; Zelditch, Lundrigan & Garland, 2004) and we might have size differences among our studied species, we performed all analysis on the residuals from a regression of shape on centroid size. Those residuals are shape values from which the effects of size have been removed (Klingenberg, 2011). We computed species mean shapes of all species in the study and performed a principal component analysis (PCA) on a covariance matrix. It is a widely used method to visualize variation within a sample and to characterize the main features of shape variation. PCA, where groups are not defined a priori, allowing us to have an insight in the shape variation of the claws among the different habitats. For the littoral species we did canonical variate analysis (CVA) and discriminant function analysis (DFA) to explore the degree of claw shape dissimilarity among habitats. Those methods separated known groups in the data and provided an ordination that maximizes the separation of group means relative to the variation within groups. DFA was performed to test the probability that a given individual was properly assigned to their a priori group. In PAST we created an unrooted neighbor-joining tree based on Mahalanobis distances derived from prior CVA to further examine the claw similarities among species and to get a good overview about claw shapes of species in different environments. For visualization of differences we produced a mean shape wireframe graph of a typical claw for each habitat (‘mangrove’, ‘mix’ and ‘rock’) in MorphoJ.

## Results

**Figure 2:**
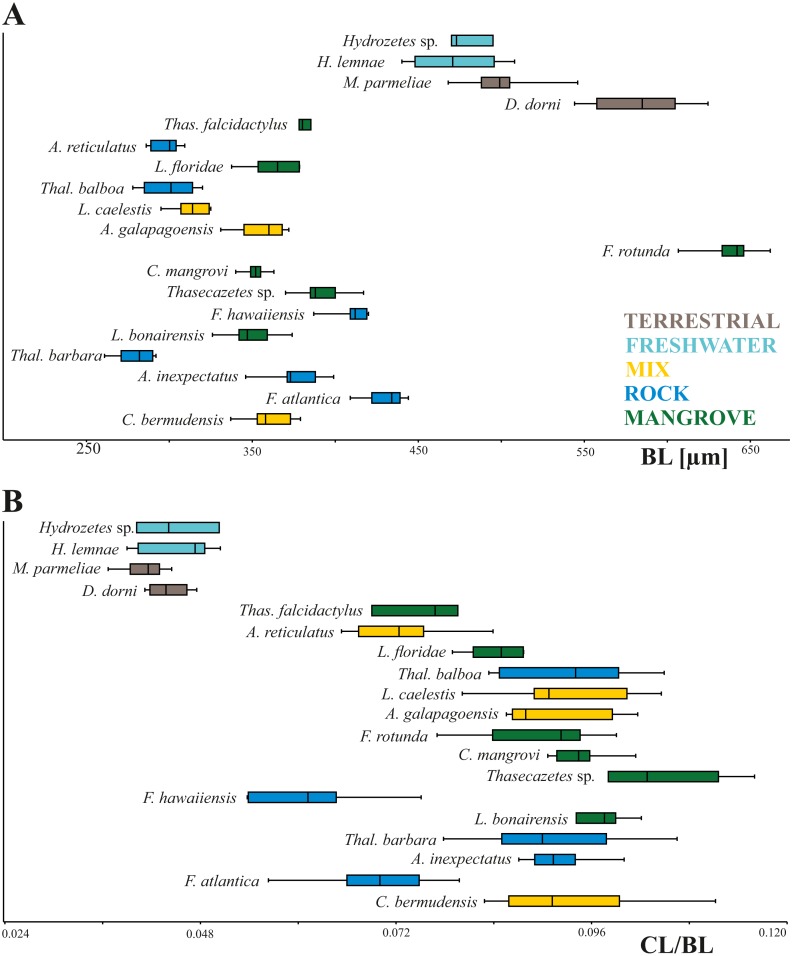
Body length and claw length for different species and habitats. Box plots of (A) body length and (B) claw length in relation to body length according to different species and habitats. Different colors mean different habitats.

In our study body length of mites ranges from 261 µm to 662 µm with *Thalassozetes barbara* as the smallest and *Fortuynia rotunda* as the largest species. Studied terrestrial and freshwater mites are nearly twice as large as littoral ones. There is no evidence for a relation between microhabitat and body size, also there does not appear to be any correlation between microhabitat and relative claw length in littoral mites (Fig. 2). Interestingly *Fortuynia rotunda* which is the largest species in terms of body length, does not show an exceptionally big claw. However, terrestrial and aquatic mite species show more diverging length ratios than littoral mite species and their claws are only half as large as the claws of intertidal species (Fig. 2).

**Figure 3:**
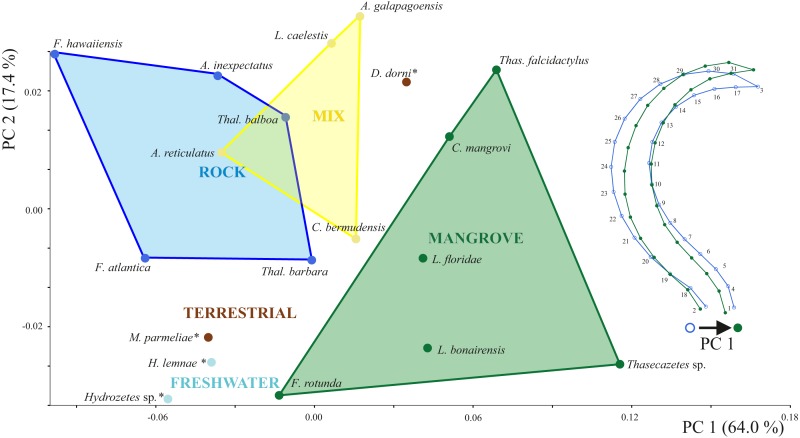
Principal component analysis of claw shapes. Scatter plot of the first two principal components (PCs), accounting for 81% of total shape variation among the 19 species means (* outgroup). Picture on the right shows shape differences corresponding to PC 1. Shape deformations according to PC 2 are given in Fig. S1.

Principal components 1 and 2 account for 71.4% of variation in the data. Species inhabiting rocks have clearly different claw shapes than those from mangroves (Fig. 3). The morphospace shows that species with a mixed microhabitat range have PC 1 scores in between the rock and mangrove inhabiting species. PC 1 represents shape differences between a slender and elongate claw at low PC1 scores, and a broad and more curved claw at high PC1 scores. In mangrove species the whole claw becomes slender and less curved. PC 2 does not show any clear separation in terms of littoral habitat or species and is characterized by a slight variation in the claw’s curvature (Fig. S1). The two terrestrial species *Dolicheremeaus dorni* and *Mycobates parmeliae* show strongly diverging mean shapes of their claws, whereas claws of the two freshwater mite species *Hydrozetes* sp. and *Hydrozetes lemnae* are similar to each other. Nevertheless, outgroup species clearly cluster outside the littoral species.

**Figure 4:**
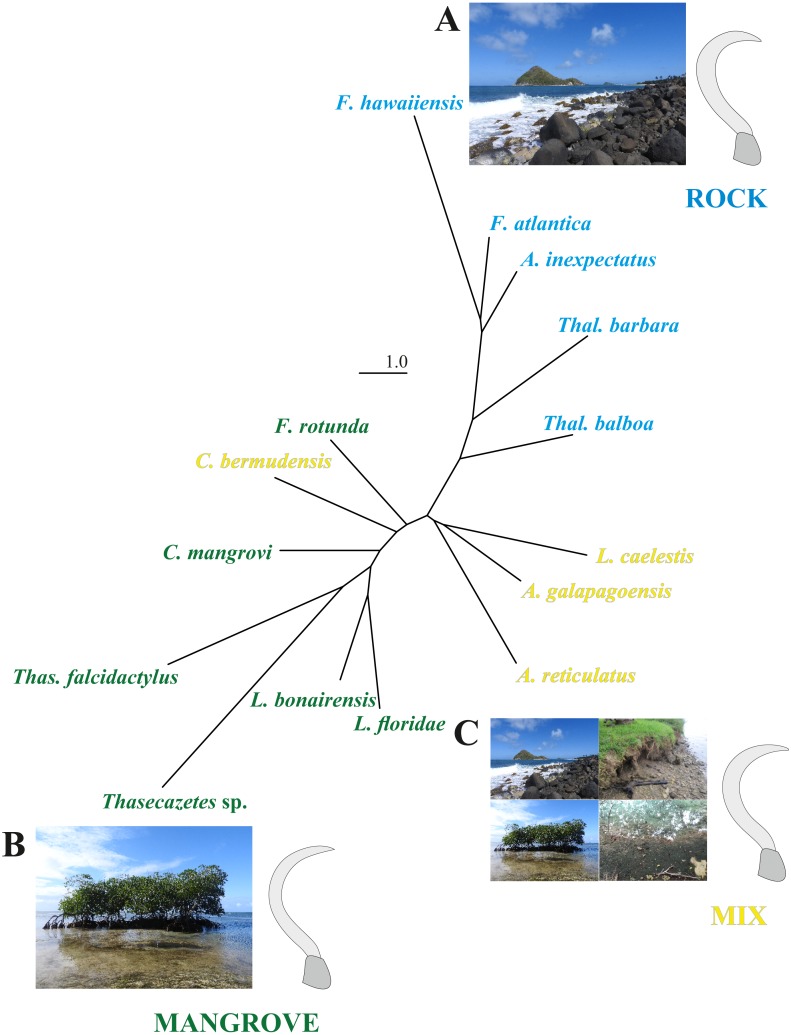
NJ tree and claw shapes according to different microhabitats. Unrooted neighbor-joining tree based on Mahalanobis distances obtained from a CVA on landmark data of the littoral mite species in three microhabitats. Photographs and mean claw shape of all specimens from microhabitat (A) rock, (B) mangrove and (C) mix.

The first two CV axes account for 61% of the shape variation among species. The scatterplot of the CV scores shows a gradient of mangrove species to rock species along the CV1, with a marked concentration of rock species at high CV1 scores (Fig. S2). Reinforcing these findings, the neighbor-joining tree based on Mahalanobis distances (Fig. 4) reveals remarkable differences between the microhabitat ‘rock’ and ‘mangrove’. Figure 4 includes pictures of the typical claw shapes for each littoral microhabitat. We can state that mite species living in the rocky habitat exhibit a more curved and broader claw and those inhabiting mangrove litter and roots show a more pointed, slender and dorsoventrally flattened claw. Euryoecious mites have an intermediate type of the “rocky” and the “mangrove” claw. Finally, DFA results show that the three habitats are well separated and differences between means are significant. For the mangrove vs. rock and the mix vs. rock comparison we get 100% correct classification (Table 2).

## Discussion

In the present study, all mites no matter from which substrate show sharp claws. Claw tips should be as sharp as possible to maximize interlocking ability (Asbeck et al., 2006) as claws tend to interlock with substrate irregularities when the roughness is larger than the tip radius (Dai, Gorb & Schwarz, 2002). Our results further demonstrate that, apart from tip sharpness, claw shape strongly correlates with microhabitat, substrate use respectively. Intertidal rock species show higher and stronger curved claws, especially in the distal part, while mangrove species possess lower and less curved claws. Higher claws are thought to be more resistant to break when subject to vertical forces, as occurs when an animal is climbing (Ribas et al., 2004). Accordingly, greater claw height is considered indicative of vertical climbers, as for example, in lizards and birds (Feduccia, 1993; Crandell et al., 2014; Ribas et al., 2004; Tulli et al., 2009). In intertidal mites, on the other hand, vertical climbing is shown equally in all microhabitat groups, they are moving vertically on rock surfaces, mangrove roots etc. However, rocky habitats are often very exposed steep environments, e.g., cliffs, with a strong surge whereas mangroves usually grow in places with flat underground and less exposition to wave action, e.g., estuaries. Consequently, mites experience stronger external forces in rocky habitats and therefore the larger heights of their claws may avoid them to be broken due to the strong wave action. Claw height is also suggested to improve the attachment ability on rough surfaces like rock (Zani, 2000) as it was shown in a varanid guild inhabiting rocky fields (D’Amore et al., 2018) and this may also apply to intertidal mites.

Our results also demonstrate that mite species inhabiting rocky habitats possess significantly stronger curved claws than species dwelling on softer substrates, like mangrove roots or leave litter. Therefore, a greater curvature of the claw may increase the clinging ability on hard, rocky substrates. Softer plant surfaces have a wide range of textures, they may be hairy or rough and thus provide good grip. Even if they are smooth, they are often covered with waxes or other secretions (Dai, Gorb & Schwarz, 2002) which may facilitate attachment. A strong curved claw is seemingly not selected for on these substrates. The ‘mix’ mite species exhibit an intermediate claw type between ‘rock’ and ‘mangrove’ which apparently allows them to dwell in a wide range of intertidal habitats. In lizards, generalist species also show intermediate positions between the high, strongly curved claws of saxicolous species and the low, less curved claws of terrestrial species (Tulli et al., 2009). Greater degrees of curvature were shown, for example, in tree climbing avians and reptiles (e.g., Feduccia, 1993; Tulli et al., 2009) whereas lower curved claws are present in ground dwelling birds (Birn-Jeffery et al., 2012) and lizards, especially in species occurring on sandy soils (Tulli et al., 2009). These results suggest that claw curvature is positively correlated with clinging ability. However, other studies (Zani, 2000; Crandell et al., 2014) found no significant relationship and proposed that claw curvature is not strongly correlated with habitat use (Tulli et al., 2009). A clear correlation between claw curvature and ecology could though be demonstrated in *Anolis cybotes,* an ecologically variable species, where individuals from rocky areas showed more strongly curved claws (Wollenberg et al., 2013), similarly to our observations.

**Table 2:** Results from DFA. Discriminant function analysis indicating claw shape differences among the three habitats of mites from the littoral habitat.

**Microhabitat**	**Hotelling’s T^2^**	*p*-value	**Right allocations**
mangrove vs. mix	433.3	0.005	84%
mangrove vs. rock	1,611.8	<0.0001	100%
mix vs. rock	726.1	<0.0001	100%

Certain ecomorphological studies (Herrel, Meyers & Vanhooydonck, 2001; Tulli et al., 2009) have shown that a strong phylogenetic signal can often obscure correlations between morphology and ecology when comparing more closely related groups. In the present study, the ecology seems to outweigh relationship as even closely related congeneric species are clearly separated because they cluster in different ecological groups. The intertidal habitat obviously poses very specific demands on the claw shape of the animals resulting in a strong correlation between morphology and ecology and the best predictors of habitat use seem to be variables related to claw height and curvature.

Our results also highlight that claws of littoral mite species are, in relation to body size, nearly twice as large as claws of typical terrestrial and freshwater aquatic species. Karasawa & Hijii (2004) already demonstrated that littoral oribatid species show relatively larger claws than arboreal species and the present data apparently support this finding. Pugh (1987) noticed that the claws of eulittoral *Ameronothrus* species are proportionally longer than those of congeneric supralittoral species. Accordingly, getting a grip and moving in the tidal surge basically requires longer claws. Among investigated intertidal mites, claw length varies to a certain extent but with no specific pattern or trend, i.e., rock species do not show longer claws than mangrove species or vice versa. Hence, not the substrate but the moving tidal water is the most significant ecological factor shaping claw length. Unfortunately, there are no comparable studies on other littoral arthropods or vertebrates yet. However, in terrestrial environments ecological factors influencing claw length may be more complex resulting in contrasting patterns. In Anoles, for example, significantly longer claws can be found in arboreal species or species that are found higher in the trees (Crandell et al., 2014) whereas in certain iguanas, on the other hand, sand-dwelling species tend to have longer claws than arboreal or saxicolous species.

Karasawa & Hijii (2004) also demonstrated that most littoral oribatid mite species are monodactyl while terrestrial taxa usually are tridactyl. Why a single claw is more favorable in the intertidal environment cannot be answered with the present data, but observations of living specimens have shown that intertidal mites are fast runners and climbers which may represent an adaptation to the fast-changing intertidal environment (Pfingstl, 2013). From a mechanical point of view, three claws may provide more grip than a single claw but detaching probably takes longer which may prevent fast movements. This may explain the lack of three-clawed oribatid mites in the littoral zone. Nevertheless, more experimental studies are needed to verify if the intertidal environment favors fast running and if this ability is linked to a single claw.

Our study is the first geometric morphometric study of any arthropod claw and provides strong evidence for a relationship between morphology and ecology. The intertidal environment harbors many other microarthropods, like predatory mites, collembola or staphylinid beetles, and the forces placed upon their claws are the same, therefore similar ecomorphological patterns may be present. Claw morphology correlates well with microhabitat suggesting that claw shape plays an important role in niche separation, at least in the intertidal environment. However, considering the large number of different terrestrial microhabitats and substrates, the same may apply to terrestrial microarthropods and their environments. Exploring the correlation between phenotype and performance in an ecological and evolutionary context is crucial to understand the adaptive nature of phenotypic traits (Crandell et al., 2014). Despite their ubiquity in most arthropod groups, claws have been overlooked and their functionality is little understood. Follow up studies would represent important next steps to unravel the functional, ecological and evolutionary significance of claw morphology in any given taxon.

## Conclusions

The present data demonstrate that claw shape of intertidal oribatid mites is strongly correlated with microhabitat use. Species dwelling on hard, rocky substrates show high (from dorsal to ventral edge) and strongly curved claws while species living on softer substrates, like mangrove litter and roots, show significantly lower and less curved claws. In comparison with terrestrial and freshwater aquatic taxa, littoral species show significantly longer claws suggesting that tidal moving water selects for remarkably long claws. These ecomorphological relationships may have played major roles in the evolutionary invasion of the littoral environment and its different niches.

##  Supplemental Information

10.7717/peerj.8488/supp-1 Supplemental Information 1 Raw data

10.7717/peerj.8488/supp-2 Figure S1 Principal component analysis of claw shapes Shape deformations according to PC 2. Differences from the negative PC 2 axes (blue) to the positive axes (green).

10.7717/peerj.8488/supp-3 Figure S2 Canonical variate analysis of claw shapes Scatter plot of the first two canonical variates (CVs), accounting for 61% of total shape variation among littoral species. Colors of species names represent microhabitats (mangrove, mix and rock).

10.7717/peerj.8488/supp-4 Table S1 Mahalanobis analysis Mahalanobis distances among littoral species from CVA

## Additional Information and Declarations

### Data Availability

The following information was supplied regarding data availability: The raw measurements and data are available in a Supplementary File. Photographs of the claws are available at Dryad: DOI https://doi.org/10.5061/dryad.0gb5mkkxc.
