# Supplementary figures and images for: Get a grip—evolution of claw shape in relation to microhabitat use in intertidal arthropods (Acari, Oribatida)
Source: PeerJ. 2020 Feb 13;8:e8488. doi: 10.7717/peerj.8488 (PMC7024575; doi:10.7717/peerj.8488)

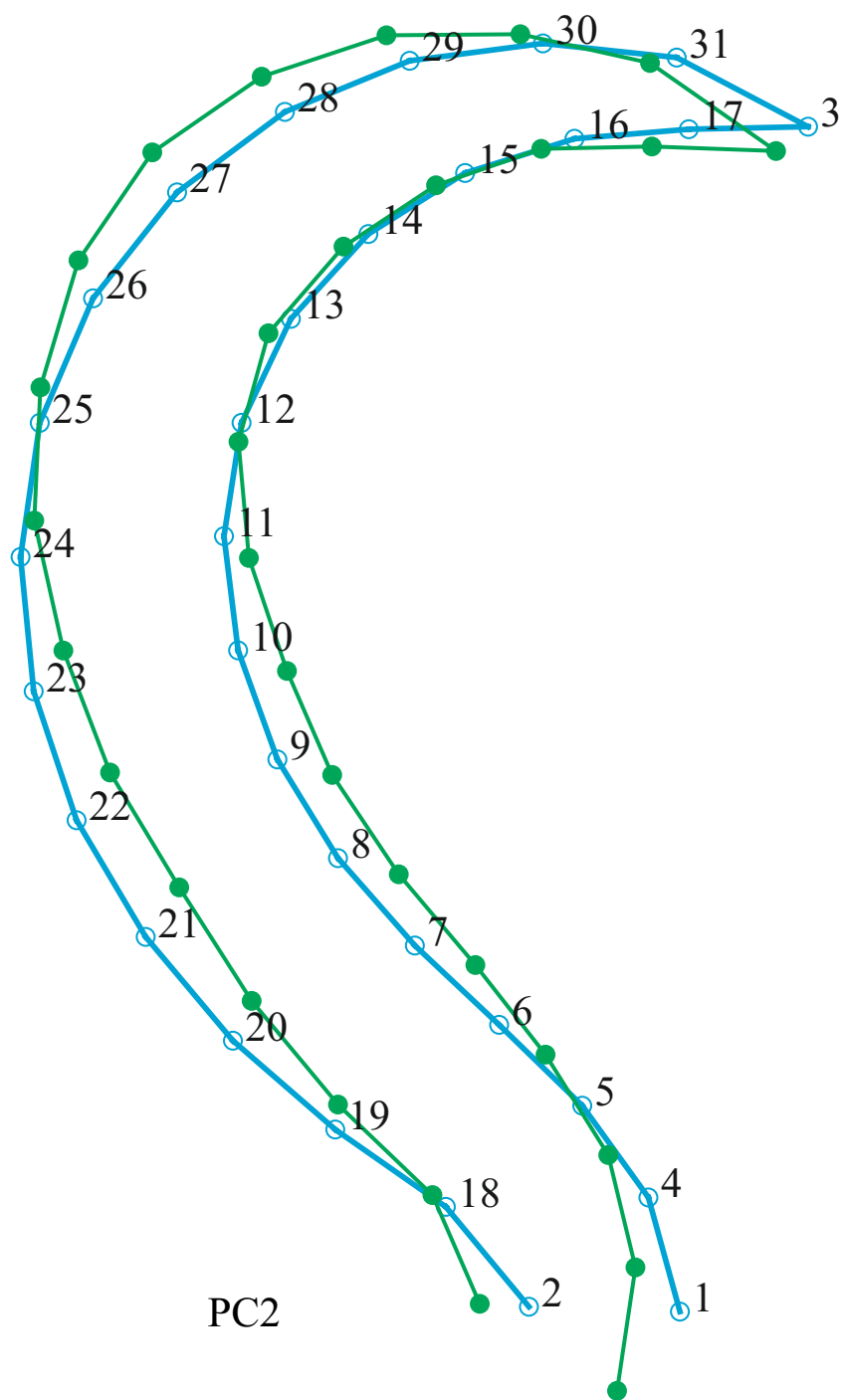

Supplement: Figure S1 — Shape deformations according to PC 2. Differences from the negative PC 2 axes (blue) to the positive axes (green). [file peerj-08-8488-s002.pdf]

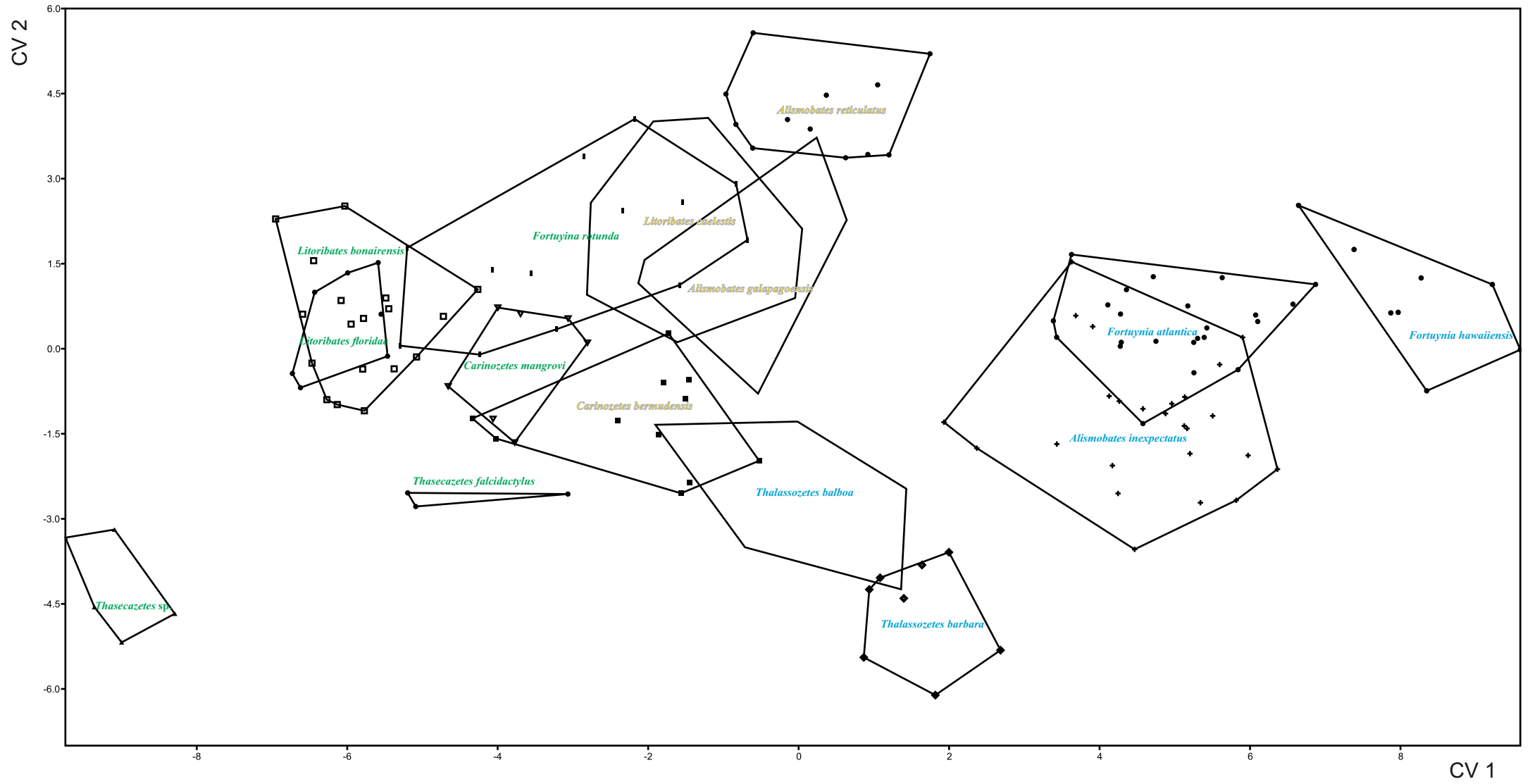

Supplement: Figure S2 — Scatter plot of the first two canonical variates (CVs), accounting for 61% of total shape variation among littoral species. Colors of species names represent microhabitats (mangrove, mix and rock). [file peerj-08-8488-s003.pdf]
