# Supplementary material for: Get a grip—evolution of claw shape in relation to microhabitat use in intertidal arthropods (Acari, Oribatida)
Source: PeerJ. 2020 Feb 13;8:e8488. doi: 10.7717/peerj.8488 (PMC7024575; doi:10.7717/peerj.8488)
Supplement: Table S1 — Mahalanobis distances among littoral species from CVA [file peerj-08-8488-s004.pdf]

Table S1. Mahalanobis distances among littoral species from CVA

	Alismobates galapagoensis	*Alismobates inexpectatus*	*Alismobates reticulatus*	*Carinozetes bermudensis*	*Carinozetes mangrovi*	*Fortuynia atlantica*	*Fortuynia hawaiiensis*	*Fortuynia rotunda*	*Litoribates bonairensis*	*Litoribates caelestis*	*Litoribates floridae*	*Thalassozetes balboa*	*Thalassozetes barbara*	*Thasecazetes falcidactylus*	*Thasecazetes* sp.
Alismobates galapagoensis	0.000	7.224	5.659	6.290	5.403	7.688	9.976	5.510	6.821	5.129	6.954	6.115	8.509	8.814	11.417
Alismobates inexpectatus	7.224	0.000	7.750	8.207	9.219	3.887	5.877	8.791	10.868	8.364	11.486	6.608	5.879	11.314	14.792
Alismobates reticulatus	5.659	7.750	0.000	7.464	7.750	7.389	9.794	5.928	8.213	6.880	8.850	8.205	9.806	10.584	13.311
Carinozetes bermudensis	6.290	8.207	7.464	0.000	5.741	8.138	11.508	5.546	6.247	7.611	7.206	6.327	7.590	7.893	9.244
Carinozetes mangrovi	5.403	9.219	7.750	5.741	0.000	9.572	13.034	5.524	5.001	6.075	5.735	7.296	8.411	7.300	8.710
Fortuynia atlantica	7.688	3.887	7.389	8.138	9.572	0.000	6.279	8.752	11.306	8.388	12.108	7.945	7.534	12.371	15.376
Fortuynia hawaiiensis	9.976	5.877	9.794	11.508	13.034	6.279	0.000	11.993	14.603	11.414	14.670	9.759	9.941	15.003	19.048
Fortuynia rotunda	5.510	8.791	5.928	5.546	5.524	8.752	11.993	0.000	5.099	6.254	5.676	7.473	8.779	9.040	9.948
Litoribates bonairensis	6.821	10.868	8.213	6.247	5.001	11.306	14.603	5.099	0.000	7.345	4.305	8.544	9.569	8.282	8.030
Litoribates caelestis	5.129	8.364	6.880	7.611	6.075	8.388	11.414	6.254	7.345	0.000	7.975	7.620	8.975	9.115	12.317
Litoribates floridae	6.954	11.486	8.850	7.206	5.735	12.108	14.670	5.676	4.305	7.975	0.000	8.102	10.352	8.549	8.610
Thalassozetes balboa	6.115	6.608	8.205	6.327	7.296	7.945	9.759	7.473	8.544	7.620	8.102	0.000	6.200	8.611	11.508
Thalassozetes barbara	8.509	5.879	9.806	7.590	8.411	7.534	9.941	8.779	9.569	8.975	10.352	6.200	0.000	9.969	12.364
Thasecazetes falcidactylus	8.814	11.314	10.584	7.893	7.300	12.371	15.003	9.040	8.282	9.115	8.549	8.611	9.969	0.000	9.776
Thasecazetes sp.	11.417	14.792	13.311	9.244	8.710	15.376	19.048	9.948	8.030	12.317	8.610	11.508	12.364	9.776	0.000
